# Factors associated with the Household Income of Persons Living with HIV/AIDS in China

**DOI:** 10.5539/gjhs.v4n3p108

**Published:** 2012-05-01

**Authors:** Xiulan Zhang, Yurong Zhang, Tamara Aleong, Tobi Baker, Esme Fuller-Thomson

**Affiliations:** 1 School of Social Development and Public Policy (SSDPP), Beijing Normal University, Beijing, China; 2 Factor-Inwentash Faculty of Social Work, University of Toronto, Ontario, Canada

**Keywords:** household income, Human Immunodeficiency Virus, Acquired Immune Deficiency Syndrome

## Abstract

This study provides a profile of 866 people living with HIV/AIDS (PLWHA) in three provinces in rural China and identifies factors associated with per-capita income in AIDS-affected households. The majority of the participants were female, married, had completed primary school, and were 30-49 years of age. Thirty percent of respondents lived in a household with at least one other HIV/AIDS patient and 15% had experienced the death of a household member due to HIV/AIDS. Therefore, health professionals should be aware of issues of grief and caregiver burnout among rural PLWHA and their families. Three-quarters of the respondents continued to work after being diagnosed with HIV/AIDS. Household per capita income was significantly higher for married individuals and those still working. Possible government and workplace policy initiatives that endeavor to increase income and mitigate the economic impact of HIV/AIDS on households are discussed.

## 1. Introduction

In 2007, approximately 700,000 people in China were HIV positive, with a prevalence rate in the general population of 0.05%. Although the percentages are relatively small, there are high infection rates among sub-populations in certain localities (State Council AIDS Working Committee Office & UN Theme Group on AIDS in China, 2007). These individuals represent approximately one in every 50 PLWHA in the world (UNAIDS, 2008).

The association between HIV/AIDS and poverty has been a recurrent theme in the literature (Cohen, 2000; Gillies et al., 1996; Oduaran, 2006; United Nations Population Fund, 2002; Whiteside, 2002). Although HIV/AIDS is not restricted to the poor, poverty assists the spread of HIV/AIDS (Whiteside, 2002). For example, risky occupations and poor coping strategies among those living in poverty increase the likelihood of contracting HIV/AIDS (Masanjala, 2007). While poverty does not directly cause HIV/AIDS, previous research clearly demonstrated a vicious cycle of poverty and disease (Bollinger & Stover, 1999; Booysen & Van der berg, 2005; Steinberg et al., 2002). AIDS has been widely documented (Marisa, 2005; Mushati, Gregson et al., 2003; Pitayanon & Kongsin, 1997; Yamano & Jayne, 2004). HIV/AIDS influences many aspects of the economy at a national, community, household and individual level. In terms of the human and social costs, the impact on household is the most serious and directs (Booysen et al., 2004; Bor et al., 2004; Madhavan & Schatz, 2007; Steinberg et al., 2002). HIV/AIDS most commonly strikes working-aged adults, the most productive segment of the economy (Barnett & Whiteside, 2002) and the main source of household income. At the end of 2007, those in the 15-49 year age group accounted for 93% (30,800,000) of the total HIV/AIDS cases in the world (33,200,000) (UNAIDS and WHO, 2007). In China, those aged between 20 and 49 years accounted for 69.9% of HIV/AIDS cases and 72.0% of the cumulative AIDS-related deaths (State Council AIDS Working Committee Office and UN Theme Group on HIV/AIDS in China, 2007). In addition to losing a productive member of their family, household members usually provide extensive caregiving to infected family members. HIV/AIDS has a stronger and longer-term impact on households than other diseases as it is a slow moving disease that can simultaneously affect several generations (Barnett & Clement, 2005).

In looking at specific household expenditures and income trends, the serious economic impact of HIV/AIDS on households can be illustrated. In China, households containing PLWHA earn approximately 45% of the per person income of households which are unaffected by HIV/AIDS (State Council AIDS Working Committee Office & UN Theme Group on AIDS in China, 2007). Not only are the households of PLWHA generally poorer than other families, household expenditures are also affected (Oni et al., 2002; Steinberg et al., 2002; Veenstra & Whiteside, 2005). Changes include an increase in expenditures on HIV/AIDS treatment and related transportation, and funeral costs and a concomitant decrease in expenditures on essentials such as food, childhood education, and housing (Bollinger & Stover, 1999; Booysen et al., 2004; Steinberg et al., 2002).

Gender plays a role in the economic impact of HIV/AIDS as a result of the division of labor between men and women determined by social and cultural norms (Marisa, 2005; Donovan et al., 2003; Yamano & Jayne, 2004). The economic impact of HIV/AIDS for families is more devastating when the infected individual is male. With the male breadwinner incapacitated, the newly female-headed households tend to have substantially lower earnings. This is due in part to the fact that female-headed households have greater difficulty maintaining a sustainable agriculture production level (Marisa, 2005). The severity or stage of an HIV/AIDS infection is directly related to the economic impact as well (Bollinger & Stover, 1999; Booysen et al., 2004; Naidu & Harris, 2005). Compared to other illnesses or to households with a living HIV/AIDS patient, a household that has experienced the death of an HIV/AIDS patient usually suffers greater economic losses. For example, the death of working aged PLWHA greatly affects their households’ financial status due to strong intergenerational dependency (Barnett & Clement, 2005). This is true in most developing countries that are seriously affected by HIV/AIDS as social welfare systems are inadequate in providing sufficient support. In China, intergenerational patterns of co-residence and support of elderly parents are the norm.

The social and health consequences of HIV/AIDS disproportionately affect those who are already disadvantaged due to their gender, socio-economic status, and other related social dimensions (UNAIDS, 2004). Compared with wealthy HIV/AIDS households, poor households have less capacity to cope with the impact caused by HIV/AIDS because of their lower expenditure capacity, their lack of producible assets, and their limited sources for financial loans (Basu et al., 1997; Drimie, 2003; Yamano & Jayne, 2004). A household’s socio-economic status prior to the introduction of HIV/AIDS is an important factor that affects household capacity to deal with the disease.

Although several studies have examined factors that associated with the economic impact of HIV/AIDS on households, the majority of these studies pay disproportionate attention to the estimation or calculation of costs (Ayieko, 1997; Booysen et al., 2004; Booysen & Van der berg, 2005; Lipton & Ravallion, 1995; Morrisa et al., 2000; Woolley & Marshall, 1994). This study intends to examine factors which influence the income of HIV/AIDS households through exploring how the economic impact varies according to household characteristics and individual traits of PLWHA.

## 2. Method

### 2.1 Study Sites

The survey was conducted in seven counties within the provinces of Henan, Anhui and Yunnan. The locations were selected based on their geographic location (Henan and Anhui: Central China; Yunnan: Southwest China), the dominant mode of HIV/AIDS transmission (Henan and Anhui: tainted blood and plasma donations; Yunnan: intravenous drug use), and high HIV/AIDS prevalence rates (State Council AIDS Working Committee Office & UN Theme Group on AIDS in China, 2007).

### 2.2 Study Population

This study used convenience sampling. In two of the provinces (Henan and Anhui), individuals with HIV/AIDS who received financial assistance targeted at PLWHA were provided with information about the study by staff who provided the social assistance. Those PLWHA who wished to participate were invited to contact study staff. In Yunnan province, that recruitment strategy was not available to us. Therefore, in Yunnan, we recruited through the health department. Staff who delivered HIV testing and health service provided the study information to PLWHA. Those PLWHA who were willing to join the research were asked to contact our study interviewers directly.

Only one PLWHA who was 18 years of age or older was eligible per household. In the 30% of households in the study which contained two or more individuals who had HIV/AIDS who were eligible and available and who had volunteered to be interviewed, we had the following protocol: First, if one of the two eligible household members with HIV/AIDs was the household head, we interviewed that person. If there was no eligible PLWHA who was the household head, we interviewed the spouse of household head. If the household head and the spouse were not eligible or available, the person (usually the parents or oldest children of the household head) who was most knowledgeable about the socio-economic status of household will be given priority to be interviewed.

A total of 866 PLWHA were recruited. The participants ranged in age from 20 to 67 years old, with a slightly higher number of female participants (52%). For one-quarter of the respondents, their HIV had reached full-blown AIDS.

### 2.3 Procedure

This survey was administered between November 2006 and February 2007 in the aforementioned provinces. The study protocol was conducted and approved by the institutional review board of the Beijing Normal University. Informed consent was obtained from all participants. After an interviewer described the study, participants could refuse to participate; respondents were permitted to withdraw from the survey at any time without penalty. Careful attention was paid to the participants’ requirements regarding the time and location of interviews due to concerns regarding confidentiality and stigma. To ensure the confidentiality of participants, names and other identifying information were available only to the researchers. After informed consent was obtained, standardized interviews were administered face-to-face by trained interviewers. The interviews lasted approximately one and a half to two hours and participants were given RMB 20 as compensation for their time. This amount is the equivalent of $3 US; approximately an average day’s wage for a peasant in these regions.

### 2.4 Measures

Household income per capita during the last year was the key variable of interest. Total household income was a sum of the following continuously measured variables: agricultural income, self-employment income, migrant income, government subsidies, relatives’ remittance, and other reported income. Income per capita was created by dividing the total household income by the number of people in the household.

The following socio-demographic variables of the interviewed PLWHA in each household were recorded: gender, age, education level (illiterate, primary school, middle school and above middle school), marital status (married and unmarried), role of PLWHA in the household (household head and other), employment status (whether continued to work after diagnosis) and clinical stage of HIV or AIDS (respondents taking Highly Active Anti-Retroviral Therapy (HAART) were considered to have AIDS; otherwise respondents were considered to have HIV). The following household variables were measured: family size (1-3, 4-5, and ≥6 people), the number of PLWHA in the household (1 vs 2 or more), and infection status of other family members.

### 2.5 Statistical Analysis

SPSS 11.5 data analysis package was used for all analyses. Household income per capita was therefore logarithmically transformed because income per capita had a skewed distribution and a multiple linear regression, which assessed household income per capita against each covariate, was run. Household income per capita and the continuous variable of age were not linearly related and therefore, age was categorized into 20-29, 30-39, 40-49, 50-59, and >60 years) and entered into the model as dummy variables. Categorical variables (gender, marital status, education level, etc.) were also transformed into dummy variables.

## 3. Results

The majority of the participants were female, married, had completed primary school, and were 30-49 years of age. One-quarter of the participants were in the AIDS stage of the disease. 52.1% of the participants were head of their household and over three-quarters of the respondents continued to work after diagnosis.

Most households reported were composed of four to five family members. Prior to the respondents’ HIV/AIDS diagnoses, 77.8% of the households were unaffected by HIV/AIDS; 15.1% of the households had at least one family member who had died of HIV/AIDS. Thirty percent of respondents lived in a household with at least one other HIV/AIDS patient. Table 1 also provides the mean household income per capita by each sociodemographic characteristic.

**Table 1:** Characteristics and Mean Income of respondents living with HIV/AIDS and their households in rural China (n=866)

	N	%	Mean of income per capita (RMB)	SD
Gender				
Male	415	47.9%	3126	3950
Female	451	52.1%	2639	2958
Age by decade, year				
20-29	67	7.7%	2830	2476
30-39	291	33.6%	2610	2110
40-49	323	37.3%	3046	4086
50-59	147	17.0%	2942	3979
≥60	38	4.4%	3216	5275
Education Level				
Illiterate	286	33.1%	2831	2956
Primary school	411	47.5%	2877	3857
Middle school	153	17.7%	2723	2593
Above middle school	15	1.7%	4981	7387
Marital status				
Married	618	71.4%	2747	3170
Unmarried	248	28.6%	3185	4130
Role of respondent in household				
Household head	451	52.1%	3178	4350
Spouse of household head	323	37.3%	2474	2024
Other	92	10.6%	2773	2388
Employment status				
Still worked before and after diagnosis	650	78.9%	2934	3648
Quit work after diagnosis	174	21.1%	2430	1872
Clinical stage				
AIDS	227	26.2%	2757	3430
HIV	639	73.8%	3197	3589
Household scale				
1-3	234	27.0%	3580	4483
4-5	455	52.5%	2691	3204
≥6	177	20.4%	2404	2302
Number of PLWHA in household				
1	608	70.2%	2926	3698
2 or more	258	29.8%	2748	2888
Were there other family member already infected with HIV before the respondent was diagnosed with HIV?				
No	669	77.8%	2926	3609
Yes, still alive	61	7.1%	2511	2068
Yes, but died of AIDS	130	15.1%	2767	3366

As shown in the linear regression analysis (Table 2), the log of household income per capita was significantly higher in households where PLWHA continued to work post-diagnosis compared to respondents who quit work after diagnosis (p<0.01). Household with more family members (≥6) had significantly lower income per capita (p<0.05). Respondents who were at the HIV stage (p<0.05) had significantly higher household incomes per capita in comparison to those with AIDS. There was a statistical trend (p=0.057) indicating that those who married had higher household income per capita. Neither gender, age, education level, number of PLWHA, role of PLWHA in the household, nor infection rates of other family members were associated with household income per capita.

**Table 2:** Regression analysis of characteristics of PLWHA on log household income per capita among PLWHA in rural China (n=816)

Characteristics	B coefficient	95% CI	*p* value
Gender			
Female	Ref	—	—
Male	-0.004	-0.082-0.074	0.913
Age by decade, year			
20-29	-0.062	-0.241-0.117	0.498
30-39	-0.069	-0.214-0.077	0.353
40-49	-0.015	-0.159-0.128	0.834
50-59	-0.063	-0.213-0.086	0.407
≥60	Ref	—	—
Education Level			
Illiterate	Ref	—	—
Primary school	-0.016	-0.080-0.048	0.627
Middle school	-0.033	-0.117-0.052	0.447
Above middle school	0.023	-0.209-0.255	0.844
Marital status			
Married	**0.079**	-0.002-0.161	0.057
Unmarried	Ref	—	—
Role of respondent in household			
Household head	-0.057	-0.178-0.040	0.298
Spouse of household head	-0.104	-0.235-0.026	0.116
Other	Ref	—	—
Employment status			
Still worked before and after diagnosis	**0.091**	0.026-0.156	0.006
Quit work after diagnosis	Ref	—	—
Clinical stage			
AIDS	Ref	—	—
HIV	**0.077**	0.015-0.139	0.015
Household scale			
1-3	Ref	—	—
4-5	-0.060	-0.129-0.008	0.085
≥6	**-0.107**	-0.191- -0.024	0.012
Number of PLWHA in household			
1	Ref	—	—
2 or more	-0.003	-0.067-0.061	0.929
Were there other family member already infected with HIV before the respondent was diagnosed with HIV?			
No	Ref	—	—
Yes, still alive	-0.002	-0.111-0.106	0.967
Yes, but died of AIDS	0.029	-0.060-0.118	0.525

## 4. Discussion

Household income per capita was associated with family size such that it was significantly lower in households with more family members. However due to the sharing of housing costs and other economies of scale (Statistics Canada, 2009), the actual standard of living of the larger families may not be quite as dire as it would first appear. Not surprisingly, households where PLWHA continued to work post-diagnosis had higher income levels compared to those households where PLWHA ceased to work. Household income per capita was significantly associated with marital status. This finding probably reflects the fact that marriage unions indicate the presence of additional income sources.

The findings suggest that those whose disease was still in the HIV stage were significantly richer than those who had full-blown AIDS, as has been found in previous research (Bollinger & Stover, 1999; Booysen et al., 2004; Naidu & Harris, 2005). In our study, 78.9% continued to work post-diagnosis; many of these individuals were receiving HAART treatment. In the asymptomatic stage of HIV/AIDS, it was found that most participants continued to work in order to financially support their households. The asymptomatic stage can last up to ten years, and as a result PLWHA can bring in a significant amount of income over this time. Even when working became more difficult, participants reported that they changed their work location or position, or decreased the intensity of their work in order to maintain employment. The fact that the vast majority were self-employed farmers facilitated their continued ability to work. Any contribution to the domestic farm is of value, even when the physical demands and hours worked must be modified due to the PLWHA’s physical impairments.

This trend of continued employment following an HIV/AIDS diagnosis contrasts with the findings of a study conducted in South Africa (Booysen & Van der berg, 2005) where participants discontinued employment after an HIV/AIDS diagnosis. This difference may be attributed to the well-developed social security system that exists in South Africa (Berg, 1997; Seekings, 2002), where high subsidies may discourage employment. In contrast, the coverage of the social security system in rural China is limited (Dong & Ye, 2003) and HIV/AIDS has had a huge impact on household productivity and income (Iya & Purokayo, 2012). It is necessary for PLWHA to continue to work to support their families as long as their health status permits. In fact, over 70% of the PLWHA continued to work post diagnosis.

In this study, gender and household income per capita were not significantly related which is, again, inconsistent with existing literature. Previous studies have found that, compared to the household income of female PLWHA, the household incomes of male PLWHA can be more seriously affected by HIV/AIDS (Marisa, 2005; Donovan et al., 2003; Yamano & Jayne, 2004). The possible explanation surrounds labor divisions and the culture of the surveyed provinces. In Henan and Anhui provinces, women play an important role in AIDS households. When male household heads who are affected by HIV/AIDS can no longer work, generally their female counterparts often take over the role of household financial supporter. Additionally, in the province of Yunnan, local custom and culture dictates that women provide financial support while men are less likely to work outside the home (Fei & Zhang, 2006). Such a labor division was also noted during this study’s fieldwork. Based on these varying facts regarding female employment, the finding that gender was not related to household income can be better understood.

Social programs and social policies need to be created to meet the demands of caregiving. This includes the need for respite and for financial, psychological and grief support. The caregiving demand on PLWHA and their family members may be particularly acute because over one-fifth of PLWHA are aged 50 and over and 30% of households consist of two or more PLWHA. Our data indicate very limited economic resources in these households and therefore limited ability to purchase respite care. Creative social service programs could provide government employees for nursing or home care support and/or fund the hiring of private assistance and/or respite care in local communities.

Psychological support and counseling service are urgently needed for households affected by HIV/AIDS. Living long-term with HIV/AIDS is a persistent challenge for PLWHA and their family members. The combination of the economic burden and stigma faced by many PLWHA underlines the importance of psychological and counseling services. Health professionals in rural China should screen individuals with HIV/AIDS to assess whether grief related support is needed. In our sample, 15% of respondents had experienced the death of a household member due to HIV/AIDS. Healthy grieving may be undermined due to the stigmatized nature of HIV/AIDS. There may also be feelings of anger at the deceased person who may have been the source of the current patient’s HIV/AIDS infection.

The findings suggest that supportive employment environments for PLWHA would be beneficial in mitigating the economic impact of HIV/AIDS on households and improving the quality of life of family members. The use of HAART has transitioned HIV into a chronic disease: Workplaces must strive to accommodate this disabling condition, where possible. During our field work, some PLWHA demonstrated their strong desire to continue to work both on and off the farm. Supportive policies should be created to help PLWHA to continue working. For example, tax preferential policies for small businesses and micro-loans for necessary production materials (e.g., sprayer, fertilizer and irrigation), would be preferred by many PLWHA rather than passively receiving cash AIDS assistance. Active employment policy not only improves household economic conditions, it also raises the self-confidence of PLWHA and improves their quality of life.

In addition, medical assistance is necessary for PLWHA. Current medical assistance focuses primarily on HARRT and has been very helpful to PLWHA. However, as HIV has changed into the chronic disease treatment, opportunistic infections has become an important part of HIV treatment. One possible consideration is to integrate the assessment and treatment of opportunistic infections into New Cooperative Medical System (NCMS) - the main rural medical care security system in China. Some rural regions have already piloted the integration of HIV treatment into the NCMS.

Findings should be interpreted with caution due to the limitations of the study: First, our recruitment strategy in two provinces was focused on those who were receiving social assistance for PLWHA and thus our sample does not represent rural Chinese PLWHA. The sample may also not be representative of PLWHA receiving public assistance, since we have no way of assessing how many of those informed of the study declined to participate. A second limitation was use of self-report to determine previous income. A third limitation was the cross-sectional nature of the survey which limits causal inferences.

Despite these limitations, this study provides valuable information regarding factors associated with per capita household income and may serve as a basis for program and policy initiatives. Future research should use representative population based samples, prospective panel studies and gather information on a greater range of factors which may influence the economic well-being of PLWHA.
